# Unpacking Prevalence and Dichotomy in Quick Sequential Organ Failure Assessment and Systemic Inflammatory Response Syndrome Parameters: Observational Data–Driven Approach Backed by Sepsis Pathophysiology

**DOI:** 10.2196/18352

**Published:** 2020-12-03

**Authors:** Nazmus Sakib, Sheikh Iqbal Ahamed, Rumi Ahmed Khan, Paul M Griffin, Md Munirul Haque

**Affiliations:** 1 Ubicomp Lab Department of Computer Science Marquette University Milwaukee, WI United States; 2 College of Medicine University of Central Florida Orlando, FL United States; 3 Regenstrief Center for Healthcare Engineering Purdue University West Lafayette, IN United States; 4 RB Annis School of Engineering University of Indianapolis Indianapolis, IN United States

**Keywords:** sepsis, MIMIC-III, SIRS, qSOFA, pathophysiology, medical internet research, medical informatics, critical care, intensive care unit, multicollinearity

## Abstract

**Background:**

Considering morbidity, mortality, and annual treatment costs, the dramatic rise in the incidence of sepsis and septic shock among intensive care unit (ICU) admissions in US hospitals is an increasing concern. Recent changes in the sepsis definition (sepsis-3), based on the quick Sequential Organ Failure Assessment (qSOFA), have motivated the international medical informatics research community to investigate score recalculation and information retrieval, and to study the intersection between sepsis-3 and the previous definition (sepsis-2) based on systemic inflammatory response syndrome (SIRS) parameters.

**Objective:**

The objective of this study was three-fold. First, we aimed to unpack the most prevalent criterion for sepsis (for both sepsis-3 and sepsis-2 predictors). Second, we intended to determine the most prevalent sepsis scenario in the ICU among 4 possible scenarios for qSOFA and 11 possible scenarios for SIRS. Third, we investigated the multicollinearity or dichotomy among qSOFA and SIRS predictors.

**Methods:**

This observational study was conducted according to the most recent update of Medical Information Mart for Intensive Care (MIMIC-III, Version 1.4), the critical care database developed by MIT. The qSOFA (sepsis-3) and SIRS (sepsis-2) parameters were analyzed for patients admitted to critical care units from 2001 to 2012 in Beth Israel Deaconess Medical Center (Boston, MA, USA) to determine the prevalence and underlying relation between these parameters among patients undergoing sepsis screening. We adopted a multiblind Delphi method to seek a rationale for decisions in several stages of the research design regarding handling missing data and outlier values, statistical imputations and biases, and generalizability of the study.

**Results:**

Altered mental status in the Glasgow Coma Scale (59.28%, 38,854/65,545 observations) was the most prevalent sepsis-3 (qSOFA) criterion and the white blood cell count (53.12%, 17,163/32,311 observations) was the most prevalent sepsis-2 (SIRS) criterion confronted in the ICU. In addition, the two-factored sepsis criterion of high respiratory rate (≥22 breaths/minute) and altered mental status (28.19%, among four possible qSOFA scenarios besides no sepsis) was the most prevalent sepsis-3 (qSOFA) scenario, and the three-factored sepsis criterion of tachypnea, high heart rate, and high white blood cell count (12.32%, among 11 possible scenarios besides no sepsis) was the most prevalent sepsis-2 (SIRS) scenario in the ICU. Moreover, the absolute Pearson correlation coefficients were not significant, thereby nullifying the likelihood of any linear correlation among the critical parameters and assuring the lack of multicollinearity between the parameters. Although this further bolsters evidence for their dichotomy, the absence of multicollinearity cannot guarantee that two random variables are statistically independent.

**Conclusions:**

Quantifying the prevalence of the qSOFA criteria of sepsis-3 in comparison with the SIRS criteria of sepsis-2, and understanding the underlying dichotomy among these parameters provides significant inferences for sepsis treatment initiatives in the ICU and informing hospital resource allocation. These data-driven results further offer design implications for multiparameter intelligent sepsis prediction in the ICU.

## Introduction

Sepsis remains one of the most elusive syndromes in medical science, which is a syndrome induced by infection and associated with biochemical, physiological, and pathological abnormalities as a result of an unregulated response from the human body [1-3]. In the United States, over 1.7 million adults are affected by sepsis, and more than 970,000 patients are admitted to hospitals because of sepsis each year. Sepsis both directly and indirectly contributes to more than 250,000 deaths annually, representing more than 50% of all hospital deaths [2,4-8]. Unfortunately, these excruciating statistics have been exacerbated over recent years, as identified in a two-decade study on US hospitalizations, costs, and disease epidemiology. These statistics reflect an 8.7% annual increase in the incidence of sepsis among hospitalized patients in the United States [5,9,10].

Besides the alarmingly increasing incidence of sepsis and associated mortality rate, the average length of stay in hospitals is considerably higher (approximately 75% higher than that reported for most other conditions) for sepsis patients in the United States, thereby increasing the burden associated with hospital utilization [10-13]. Furthermore, the Agency for Healthcare Research and Quality [14] reported that the average length of stay for patients with sepsis dilated compellingly in 2013, and there was a distinct proportion of patients with severe sepsis cases, including 4.5 days, 6.5 days, and 16.5 days of hospitalization for sepsis, severe sepsis, and septic shock, respectively, according to the systematic inflammatory response syndrome (SIRS) criteria. Moreover, although accounting for 3.6% of hospital stays, sepsis-related care represents 13% of total US hospital costs, resulting in hospital expenses exceeding US $24 billion in 2013. Not surprisingly, in 2013, the cost associated with sepsis management ranked the highest among the admissions for all diseases and medical conditions, followed by osteoarthritis at US $17 billion and childbirth (medical condition) at US $13 billion [15-17]. At present, the hospital costs associated with sepsis still rank first, and sepsis care currently requires more than twice the resources required for other medical conditions [18]. These costs are also expected to be exacerbated in the near future, and will likely approach a 3-fold increase compared to those of other admissions [3,19,20].

This notable increase in mortality rate and annual health care expenditure (affected by the increased length of stay) has made sepsis treatment and research a critical domain in medical internet research and medical informatics, resulting in a recent surge in the related literature [21-24]. Studies have shown that improved and effective methods of early sepsis identification can substantially reduce the severity and epidemiological burden of sepsis in the United States [24-29]. In addition, several authors have recommended that identifying the prevalent risk factor(s), followed by an instant diagnosis, can reduce the cost in treatment workflow, and further scale down the mortality rate for patients with sepsis to some extent [26,30-33]. However, most of these studies have only concentrated on one risk factor at a time for the clinical assessment of sepsis, thereby limiting the probability for sepsis detection as it requires complex reasoning and implications. In many cases, it is apparent that the results are sensitive to subtle variations in definition(s) of sepsis, as well as subjective suspicions of physicians [21,22,34-36].

The recent major release of Medical Information Mart for Intensive Care (MIMIC-III, Version 1.4) is an extensive, single-center, and comprehensive database comprising information pertaining to patients admitted to the critical care units at Beth Israel Deaconess Medical Center in Boston, Massachusetts, including vital signs, laboratory measurements, observations and notes charted by care providers, imaging reports, fluid balance, medications, procedure codes, diagnostic codes, and hospital length of stay [17,21,37,38]. MIMIC-III is a multidisciplinary collaborative effort of the Laboratory for Computational Physiology at MIT, Computer Science and Artificial Intelligence Laboratory at MIT, and Information Systems Department at Beth Israel Deaconess Medical Center. The underlying motivation behind this collaboration is to assure reproducibility and improve the quality of data-driven medical informatics research. The salient features of MIMIC-III (Version 1.4) include that it is the only freely accessible critical care database of its kind in the United States that promotes analysis without additional restriction after accepting the data use agreement.

Furthermore, a critical care dataset with detailed individual patient care information spanning more than a decade empowers medical informatics research and pedagogy around the world. MIMIC-III (Version 1.4) contains data from 58,976 hospital admissions for patients admitted to the critical care units from 2001 to 2012. Personal information is removed, and the original records are shifted and reformatted to ensure that the data are not identifiable to human patients. The database comprises 26 tables linked by identifiers for corresponding patients. Each of the tables is a spreadsheet including information on patient hospital stays and the physiological data collected in the intensive care unit (ICU), along with data dictionaries to explain the observational context. MIMIC-III (Version 1.4) allows for a variety of data forms, ranging from text interpretations for radiology images to time-stamped physiological measures [21,37]. This open and unrestricted nature of extensive health care data allows for clinical studies to be improved and reproduced in ways that would not otherwise be possible [39]. Hence, MIMIC-III (Version 1.4) can facilitate exploratory and data-driven studies on sepsis, its diagnosis, and treatment in the ICU [17,21].

Sepsis was first formally defined by a 1991 consensus conference as a SIRS to infection in the host [1,40]. According to the then-prevailing definition, sepsis associated with organ dysfunction was referred to as severe sepsis, and severe sepsis followed by sepsis-induced persisting hypotension despite adequate fluid resuscitation was termed as septic shock. Subsequently, considering the limitations of 1991 consensus conference definitions, the 2001 task force extended the list of diagnostic criteria for sepsis [41]. Despite discrepancy in the 1991 interpretation, the 2001 task force could not offer an alternative definition due to lack of supporting evidence; therefore, the sepsis definition remained mostly unchanged from 1991 to 2016 [41,42]. In 2016, a task force comprising experts of sepsis pathobiology, pathophysiology, epidemiology, and clinical trials convened by the Society of Critical Care Medicine along with the European Society of Intensive Care Medicine revised the definition of sepsis and septic shock.

The substantial advances observed in pathobiology, epidemiology, immunology, and intervention management motivated efforts to reexamine the interpretation of sepsis. The definition devised by the 2016 task force has since been supported by 31 international sites [1]. Singer et al [1] concluded that it is necessary to change the perception about sepsis to establish a more reliable predictive indicator of mortality and impact in the survivability of patients. Consequently, the SIRS-based definition was replaced by the quick Sequential Organ Failure Assessment (qSOFA) criteria. The qSOFA suggests three criteria to evaluate patients who are more likely to have a poor outcome due to sepsis: hypotension, altered mental status, and high respiratory rate [21]. In addition to qSOFA, the sepsis-3 definition (given that this was the third updated definition of sepsis) includes the Sepsis-related Organ Failure Assessment (SOFA) for making a sepsis diagnosis. Albeit not substantially, SOFA provides better predictive accuracy with greater consistency compared to qSOFA. However, the intricacy and time-consuming lab tests involved in SOFA have remained poorly understood outside the critical care community since the definition was updated in 2016.

As sepsis is still perceived as a spectrum disease that subsequently ends in organ dysfunction, septic shock is a crucial juncture for multiparameter intelligent sepsis prediction in the ICU. However, we here focus on sepsis defined according to SIRS and qSOFA. We adopted a data-driven approach using MIMIC-III (Version 1.4) to offer unique contributions to the field. First, we aimed to unpack the most prevalent SIRS and qSOFA criteria. Second, we evaluated the most prevalent sepsis scenarios based on SIRS and qSOFA criteria. Third, we investigated the dichotomy among SIRS and qSOFA criteria to establish underlying statistical relations among these predictors, with design implications for predictive modeling. Quantifying the prevalence of the qSOFA criteria (in comparison with SIRS) and understanding the underlying dichotomy of these parameters have important implications for sepsis treatment initiatives in the ICU and for informing hospital resource allocation. Hence, this study has potential to improve preventable deaths from sepsis.

## Methods

### Theoretical Background

#### Sepsis Pathophysiology

Sepsis—commonly interpreted as a spectrum disease—ranges from milder symptoms and ends in septic shock, followed by multiple organ dysfunction syndromes. This entire spectrum begins with the introduction of pathogens in the blood vessels, such as gram-positive or gram-negative bacteria, fungi, viruses, and parasites. The appearance of pathogens in the blood vessels makes them no longer sterile; when the white blood cells confront these infective materials (pathogens), they become activated. Consequently, more white blood cells are called in to the site of infection to eradicate the pathogens. Generally, these infective materials exist outside in the interstitial tissue rather than in the bloodstream. Therefore, to access the infective materials and eradicate them, the white blood cells release substances such as nitric oxide. Three events occur once these substances interact with the blood vessels. First, the diameter of the blood vessel expands, resulting in vasodilation. The vasodilation reduces the localized systemic vascular resistance and affects the speed of the blood flow, including the blood flow in the infected area. Second, the permeability of the blood vessels increases so that the immune system can confront the peripheral infective material easily. In the context of this paper, blood pressure—in the mathematical sense—is considered to be the product of cardiac output and systemic vascular resistance, thus affecting tissue perfusion. Hence, the lower the systemic vascular resistance, the lower the blood pressure, and consequently tissue perfusion is reduced [43,44].

The decrease in tissue perfusion is further exacerbated by the increased permeability of the blood vessels since the fluid can reach out and build around the tissue, which eventually makes it challenging for oxygen to diffuse through the fluids and access the cells. This exacerbated tissue perfusion is the cardinal reason behind the shock. Third, when the white blood cells interact with the pathogens, they release lytic enzymes as well as reactive oxygen species to eliminate the infective materials. These enzymes damage not only the pathogens but also the blood vessels to some extent, resulting in serious complications. When the blood vessels are ruptured, proteins are released to cause clotting as a patch due to coagulation factors in the blood. This may initially preclude the blood from spilling into the extravascular space; however, over time, some of these clots can break off into the bloodstream to allow the blood to spill out of the blood vessels, resulting in disseminated intravascular coagulation. Since this complication is disseminated throughout the body, the damaging enzymes and cytokines associated with different immune molecules may also cause damage to the blood vessels in the lungs. Damage and rupture in all of the blood vessels in the lungs seriously affects oxygen absorption into the bloodstream, resulting in acute respiratory distress syndrome. This can lead to severe respiratory distress since the respiratory system can no longer pull in oxygen into the bloodstream from the environment. In response, the human body initially pushes to increase the cardiac output to compensate for the decreased systemic vascular resistance so as to maintain blood pressure. However, if remained untreated, the septic shock will persist and the cardiac output will eventually start to be depressed, resulting in a serious decrease in cardiac output [43-46]. These pathophysiological incidents caused by sepsis are reflected in several physiological parameters as clinical clues, hence commonly named as symptom distributives. Although highly elusive in nature, the entire purpose of the sepsis-3 and sepsis-2 definitions is to capture the underlying symptom distributives that are the most relevant.

#### Bedside Monitoring: qSOFA vs SIRS

Sepsis, unlike most other human diseases, is not a specific disease entity but rather a syndrome consorted with an ambiguous pathobiology and the absence of gold-standard diagnostic tests for assessments [1,21]. Therefore, numerous endeavors have been made to capture the pathobiology, pathophysiology, and epidemiology of sepsis to explain the syndrome. An initial definition of sepsis (sepsis-1) was introduced at the 1991 Consensus Conference that described sepsis as SIRS [21,40]. Addressing the limitations of sepsis-1, the 2001 task force extended the list of diagnostic criteria for sepsis (sepsis-2), based on SIRS, with the following four criteria: fever or hypothermia (body temperature>100.4°F or <96.8°F), tachypnea (respiratory rate >20 breaths/minute), tachycardia (heart rate >90 beats/minute), and white blood cell count >12,000/mm^3^ or <4000/mm^3^ (or >10% immature bands) [47]. In particular, sepsis-2 interprets sepsis as a cascaded disease that is primarily diagnosed as SIRS, followed by sepsis, severe sepsis, and septic shock. At the very end of the spectrum, patients may experience multiple organ dysfunction syndrome, an incurable stage of sepsis. Table 1 lists the parameters and cascaded development of sepsis as per the SIRS criteria. However, this definition failed to distinguish sepsis from the other uncomplicated infections and diseases that exhibit identical criteria, and indispensably failed to define what sepsis really is [1]. The task force also coined definitions for *severe sepsis* and *septic shock*, interpreting *severe sepsis* as sepsis complicated by organ dysfunction and *septic shock* as sepsis-induced hypotension persisting despite sufficient fluid resuscitation [47].

**Table 1:** Systemic inflammatory response syndrome (SIRS) criteria for sepsis definition.

Parameters/Criteria		Phases of syndrome development
**Criterion 1**: Body Temperature		
	>100.4°F or <96.8°F	**Phase 1**: SIRS ≥ 2 criteria
**Criterion 2**: Respiratory Rate		
	>20 breaths/minute (or PaCO_2_ <32 mmHg)	**Phase 2**: Sepsis (SIRS + suspected or confirmed infection)
**Criterion 3**: Heart Rate		
	> 90 beats/minute	**Phase 3**: Severe sepsis (sepsis + organ dysfunction)
**Criterion 4**: White blood cell count		
	>12,000/mm^3^ or <4000/mm (or >10% bands)	**Phase 4**: Septic shock (severe sepsis + persistent hypotension)
**Final Phase**: Multiple Organ Dysfunction		
	Reported ≥ 2 organs failing	

With significant advancements in the understanding of sepsis pathophysiology and pathobiology, after nearly two decades, a new definition of sepsis was proposed at the Third International Consensus in 2016 [1]. Currently, sepsis (sepsis-3) is defined as a syndrome pertaining to a life-threatening organ dysfunction introduced by a dysregulated host response to a microorganism. According to the definitions of sepsis-3, the SOFA score (criteria) is used in the ICU to determine the extent of a patient’s organ functions (dysfunction) [1]. In addition, sepsis can be promptly identified for an individual with a suspected infection at bedside using the qSOFA (sepsis-3) score. qSOFA requires satisfying at least two of the following criteria to determine that a patient is likely to have poor outcome due to sepsis [21]: respiratory rate ≥22 breaths/minutes, altered mental status (≤13 on the Glasgow Coma scale), and low blood pressure (≤100 mm Hg).

With the goal of leveraging the greater consistency of sepsis-3 in clinical trials and epidemiologic studies, several predictive machine-learning models were developed using the qSOFA parameters. Khwannimit et al [48] found that the qSOFA score showed higher prognostic accuracy for mortality and organ failure compared with SIRS criteria. Moreover, in predicting mortality and ICU-free days, qSOFA rendered considerably better discrimination in comparison with SIRS [49]. Donnele et al [50] and Hwang et al [51] provided substantial evidence to support employing SOFA and qSOFA in the ICU sepsis diagnosis and treatment workflow over SIRS criteria. However, numerous studies implied conflicting results, and asserted that qSOFA manifests inconsistent performance in mortality prediction [21]. Several studies reported that qSOFA showed poor sensitivity and inconsistent precision in the predictive models [49,51,52]. Although counterintuitive to some extent, Haydar et al [49] and Fernando et al [52] indicated that qSOFA took much longer in the patients’ trajectory in comparison with SIRS to identify patients with sepsis, which further delayed the initiation of medical interventions in the ICU, and thereby subjected the patients to a higher risk of developing septic shock and multiple organ dysfunction.

Considering these stark contrasts in the results (reflected by evaluation metrics such as accuracy, sensitivity, precision, and G-mean) of predictive modeling using SIRS and qSOFA parameters, in this study, we decided to take a step back and have a more in-depth look at the qSOFA and SIRS parameters, and their underlying attributes and interrelations. Multicollinearity among parameters often intensifies the tension between optimization and generalizability, and eventually leads to model overfitting, which in turn hampers the generalizability of discriminant functions [53]. Moreover, model overfitting indicates that a small deviation in the input data can result in considerable, and sometimes aberrant, changes in the model, even leading to changes in the sign of parameter estimates [21,53]. Table 2 compares the SIRS and qSOFA criteria, highlighting the changes brought in with sepsis-3 from sepsis-2 throughout all of the cascaded steps.

**Table 2:** Comparison of sepsis-2 and sepsis-3 criteria.

Stage	Sepsis-2 criteria (SIRS^a^)	Sepsis-3 criteria (qSOFA^b^)
Sepsis	Suspected or confirmed infection + SIRS	Suspected or confirmed infection + qSOFA score ≥2
Severe sepsis	Sepsis + organ dysfunction (lab markers, including hypoxia, hypotension, elevated lactate)	Category removed
Septic shock	Severe sepsis + persistent hypotension (after adequate fluid resuscitation)	Sepsis + vasopressors to maintain mean arterial pressure ≥65 mmHg + serum lactate level >2 mmol/L

^a^SIRS: systemic inflammatory response syndrome.

^b^qSOFA: quick Sequential Organ Failure Assessment.

### Data and Research Design

We used MIMIC-III (Version 1.4), a publicly available ICU patient database [1], for this study. The data, ranging from 2001 to 2012, involves 58,976 distinct hospital admissions. For the purpose of our study, we used the parameters of the qSOFA as well as SIRS to identify all ICU patients who had been diagnosed with sepsis or were most susceptible to the disease. We then analyzed the qSOFA and SIRS parameters of these identified sepsis patients, or the patients who had undergone sepsis screening, to study their intrarelationship. In our population, 1994 hospital admissions resulted in a diagnosis of sepsis among 58,976 overall admissions from 2001 to 2012. Among these 1994 patients, the mortality rate was 21.11% (n=421 deaths).

The selection criteria included identifying the unique key for the critical parameter records and omittable parameters that we deemed to be bias-free for the purpose of this study, such as patient gender, data storage time, and deidentified date of birth in the case of sepsis. During research design and data wrangling, we confronted missing data and outlier values that were not biologically reasonable, albeit not for a considerable amount of records. This modicum amount of unexpected data points opened up the possibility of two distinct research designs. First, we could ignore the observations that have such data point(s) because they are of negligible number compared to the total observations available. Second, we could follow the conventional central-value imputation or multiple imputations by chained equations to handle the missing data. A multiblind Delphi process, convened by Ubicomp Lab of the Department of Computer Science at Marquette University and Regenstrief Center for Healthcare Engineering at Purdue University, came to the decision that ignoring the observations that have such unexpected data point(s) will be more suitable for the purpose of this study, which requires avoiding imputation bias. Moreover, outlier values that are not biologically reasonable were excluded, considering them as mistaken data entries in the ICU [21].

**Figure 1:**
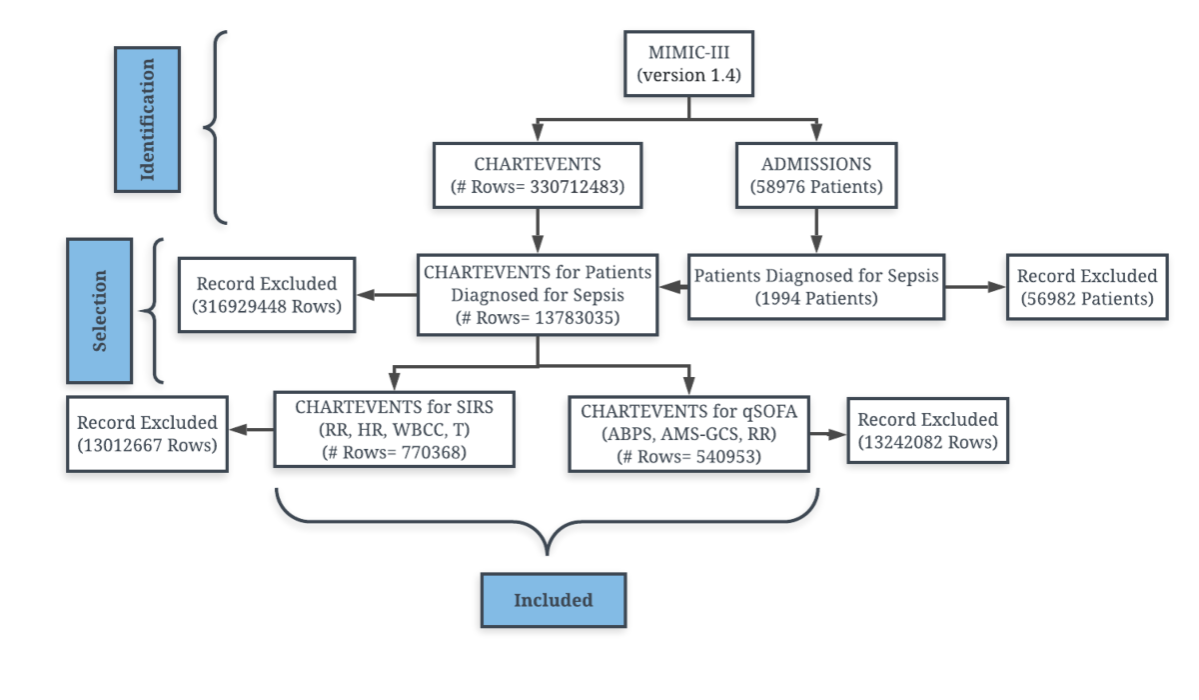
Outline of research design.

To determine the prevalence and dichotomy of the qSOFA and SIRS parameters, we identified 13,783,035 patient records (Chartevent) from 330,712,483 records (Chartevent) available in MIMIC-III (Version 1.4), which are unique for each Hospital Admission ID and chart time and pertaining to patients who had received a sepsis diagnosis. Then, to identify the most prevalent qSOFA and SIRS criteria, we selected 540,953 and 770,368 patient records for SIRS and qSOFA, respectively (in which respiratory rate was common in both cases). Figure 1 summarizes the research design in a simple flow chart.

To assure the consistency and interpretability of the results while determining the most prevalent sepsis scenario, our selection criteria only filtered within chart times for which we had observations for all three qSOFA parameters since the observation frequency varies with the parameters based on the intricacy involved in measurement. For instance, observations for altered mental status (based on the Glasgow Coma Scale) are less frequently recorded than those of the respiratory rate. More importantly, since sepsis is a spectrum disease, studying and comparing the observations for different parameters at different record times for a particular patient can confound the result and its interpretability. For the same reason, studying the parameters that are observed at the same time can capture the patient’s disease trajectory more consistently. For determining the most prevalent sepsis scenario for SIRS, our selection criteria only filtered within chart times for which we had observations for all four parameters (temperature, heart rate, respiratory rate, and white blood cell count). The white blood cell count observations are considerably less frequent compared to the other three parameters of SIRS, and therefore observations considered for the SIRS criteria are substantially reduced compared with those considered for the qSOFA criteria.

We further addressed two possible sources of selection bias. First, it is intuitive that the longer the patient stays in the ICU, there will be more observations available for that particular patient. We considered that this may influence the results of our study to some extent if there are considerably more patients with a longer length of stay. Second, when evaluating the respiratory rate for ICU patients, there may be a possible blend in the data between patients with intubated breathing and natural breathing. However, the possibility of these two selection biases also provided an opportunity to test the intrageneralizability of the results of this study (both for qSOFA and SIRS). Therefore, in the second phase of this study, we dissected our data for only the first observations of each hospital admission.

This research design is grounded in statistical theory such that the results can help in developing multiparameter intelligent sepsis prediction or treatment models that require predictors exhibiting the least or no collinearity.

## Results

### Statistical Distributions: qSOFA and SIRS

The means (SD) and median (IQR) values for qSOFA and SIRS parameters in each phase of the study are presented in Table 3. In the first phase of the study, with respect to the qSOFA criteria, we analyzed the distributions of systolic arterial blood pressure, Glasgow Coma Scale score, and respiratory rate. For the SIRS criteria, in the first phase we analyzed the distribution of heart rate, respiratory rate, temperature, and white blood cell count. In the second phase, we only considered the first observation of each hospital admission for each parameter.

**Table 3:** Statistical distributions of parameters for quick Sequential Organ Failure Assessment (qSOFA) and systemic inflammatory response syndrome (SIRS).

Parameter		Phase 1: Entire patient trajectory		Phase 2: First observation only	
		Mean (SD)	Median (IQR)	Mean (SD)	Median (IQR)
**qSOFA**					
	SABP^a^ (mmHg)	116.4 (24.78)	114.0 (100-131)	106.7 (37.62)	110.0 (96.0-126.0)
	GCS^b^	11.17 (3.66)	11.00 (9-15)	11.53 (4.32)	14.00 (8-15)
	RR^c^ (breaths/min)	21.07 (6.52)	21.00 (17-25)	20.48 (6.16)	20.00 (16.00-24.00)
**SIRS**					
	HR^d^ (beats/minute)	89.1 (18.61)	87 (76-100)	95.58 (20.76)	94.00 (80.00-109.00)
	RR (breaths/minute)	21.07 (6.52)	21.00 (17-25)	20.48 (6.16)	20.00 (16.00-24.00)
	BT^e^ (°F)	98.37 (1.57)	98.30 (97.30-99.30)	98.25 (2.01)	98.20 (97.00-99.50)
	WBC^f^ count (/mm^3^)	13.14 (7.30)	11.70 (8.10-16.70)	14.34 (8.28)	12.80 (8.50-18.90)

^a^SABP: systolic arterial blood pressure.

^b^GCS: Glasgow Coma Scale.

^c^RR: respiratory rate.

^d^HR: heart rate.

^e^BT: body temperature.

^f^WBC: white blood cell.

**Figure 2:**
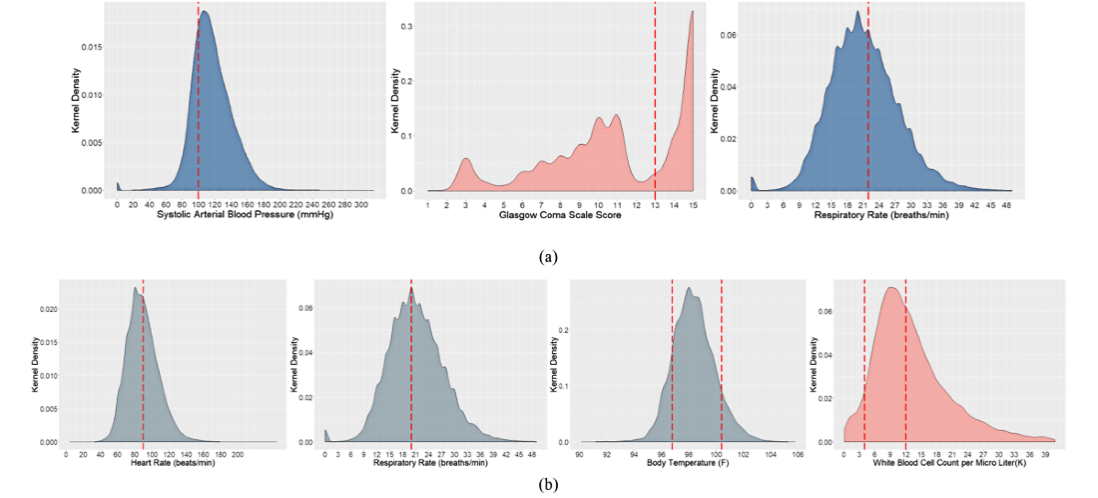
Kernel density estimation distribution of (a) quick Sequential Organ Failure Assessment (qSOFA) and (b) systemic inflammatory response syndrome (SIRS) parameters to understand the prevalence of each parameter.

Kernel density estimation distributions for the qSOFA criteria (systolic arterial blood pressure, altered mental status in Glasgow Coma Scale, and respiratory rate) and SIRS criteria (heart rate, respiratory rate, temperature, and white blood cell count) are depicted in Figure 2 to investigate the most prevalent sepsis parameter. Visual statistics demonstrated that most of the patients’ observations did not meet the qSOFA criterion for systolic arterial blood pressure (Figure 2a). The distribution for systolic arterial blood pressure implies that most of the observations were in the range of 100-125 mmHg, which is in the healthy range from the clinical point of view. Similarly, the Glasgow Coma Scale distribution (Figure 2a) indicated that a significant portion of these observations were in the safe zone (15 and 14). However, as the Glasgow Coma Scale ranges from 1 to 15, and the domain of consideration for the not-safe zone (qSOFA, 1-13) and the domain of consideration for the safe zone (14-15) are significantly disproportionate, the visual analytics may be confusing for an accurate interpretation. In the case of respiratory rate (Figure 2a), it is critical to interpret whether or not the majority of the observations met the qSOFA criterion, although it is evident that most of the data ranged between 15 and 24 breaths/minute. From the clinical point of view, at a resting state, a respiratory rate observation of 12-20 breaths/minute is considered to be healthy.

For the SIRS criteria (Figure 2b), the distribution for heart rate observations was less confounding using visual analytics in inferring prevalence, as more of the kernel density was below the criterion margin (90 beats/minute), which indicates the presence of more healthy observations. In the case of respiratory rate measurement, it is worth mentioning that the cutoff for the SIRS criteria is different than that of the qSOFA criteria. For SIRS criteria, the criterion cutoff is 20 breaths/minute, and anything above that level is considered as tachypnea. It is visually discernible that as the cutoff shifted left (from 22 to 20) for SIRS, more patient observations met the sepsis criteria. The distribution for body temperature can be interpreted as a band: the observations inside two temperature cutoffs indicate the density of the healthy observations, and they represented a significant portion of the distribution. In the case of white blood cell count, as the domain of consideration for the not-safe zone and the domain of consideration for the safe zone were significantly disproportionate, the visual analytics may be confusing to imply prevalence. However, we can infer that the majority of observations met the SIRS criteria.

In the following subsections, we provide an explicit numerical interpretation to better understand the prevalence and underlying statistical relation between the predictors.

### Patients’ Entire Trajectory for qSOFA

**Figure 3:**
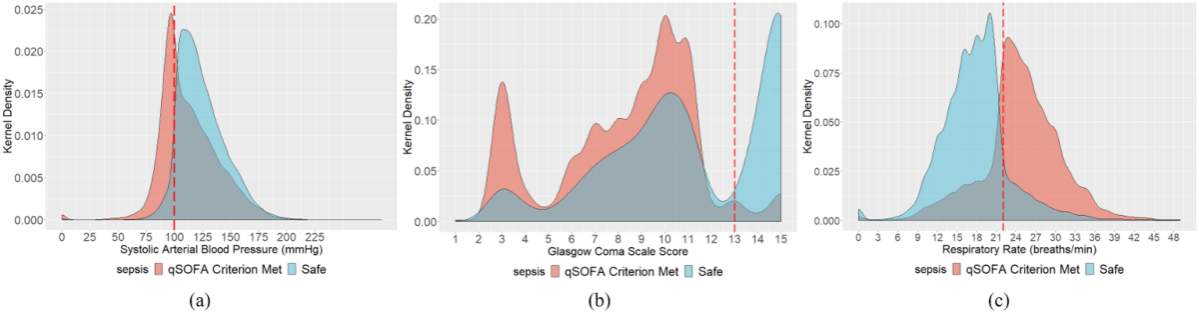
Kernel density estimation distribution of quick Sequential Organ Failure Assessment (qSOFA) parameters for both safe and qSOFA criterion-met observations to identify the prevalent qSOFA parameters.

The kernel density estimation distribution of qSOFA parameters for both safe and qSOFA criterion–met observations are presented in Figure 3 to better understand the prevalent qSOFA parameters. Overall, 25.12% of the systolic arterial blood pressure observations, 59.28% of the Glasgow Coma Scale measurements, and 45.11% of the respiratory rate observations met the respective qSOFA criterion. It is intuitive from the qSOFA criteria that determination of the most prevalent criterion from observational studies would help practitioners and researchers in further factorial experiments. This observational study entirely relied on passive retrospective observations without assigning any further treatment. The results suggest that altered mental status is the most prevalent qSOFA criterion experienced in the ICU. We further addressed a nearly double-barreled question: what is the most prevalent sepsis scenario in the ICU? We found that 28.19% of the observations (when three measurements were available at the same time) showed a two-factored qSOFA of high respiratory rate and altered mental status (among 3C_3_+3C_2_=4 possibilities), resulting in this pair identified as the most prevalent qSOFA (sepsis-3) scenario in the ICU. Notably, no sepsis is another possible scenario besides these four possible qSOFA scenarios in the ICU (which is also true for our observations).

**Figure 4:**
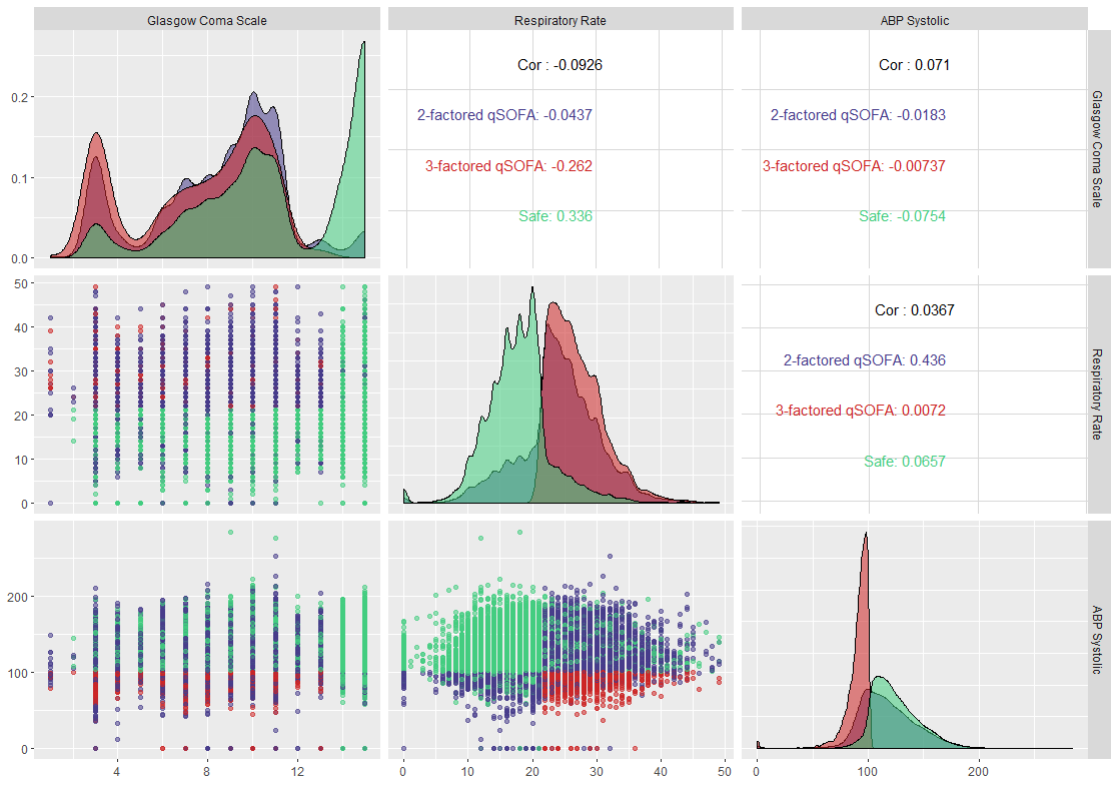
Facet grid illustration of sepsis-3 (qSOFA) parameters to capture the underlying relationship between parameters and the most prevalent sepsis scenario in the intensive care unit. qSOFA: quick Sequential Organ Failure Assessment.

Figure 4 shows a facet grid plot of the qSOFA parameters to capture the most prevalent sepsis scenario and the underlying dichotomy among the parameters. This plot has multiple implications; however, the most obvious is the comparison of the Pearson correlation coefficients (absolute) of each of the qSOFA parameters’ pairs. The absolute Pearson correlation coefficients for respiratory rate-Glasgow Coma Scale measurement, Glasgow Coma Scale measurement-systolic arterial blood pressure, and respiratory rate-systolic arterial blood pressure pairs were 0.09, 0.07, and 0.04, respectively. These insignificant correlation coefficients nullify the possibility of any linear correlation among the qSOFA parameters, thereby ensuring that multicollinearity does not exist between the parameters and further advocates for the dichotomy among them. Understanding this relationship can help in developing predictive models, as it implies that the overdetermined system involved in the modeling is a full-ranked matrix (ie, not rank-deficient). However, the lack of multicollinearity cannot guarantee that two random variables are statistically independent. Moreover, based on its pathophysiology, sepsis is a spectrum disease, and therefore one predictor may influence another during the development of sepsis and septic shock.

### Patients’ Entire Trajectory for SIRS

**Figure 5:**
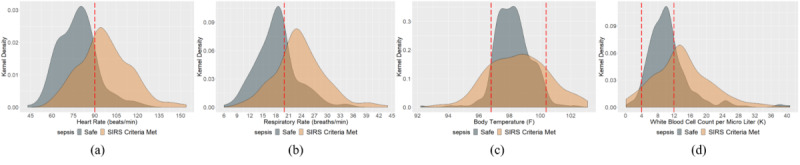
Kernel density estimation distribution of systemic inflammatory response syndrome (SIRS) parameters for both safe and sepsis criterion–met observations to identify the prevalent SIRS parameters.

Figure 5 shows the kernel density estimation distribution of SIRS parameters for both safe and SIRS criterion–met observations to understand the prevalent SIRS parameters. We found that 43.30% of the heart rate observations, 50.89% of the respiratory rate observations, 23.08% of the body temperature observations, and 53.12% of the white blood cell count observations met the respective SIRS criterion. Although both the white blood cell count and respiratory rate had a significant prevalence in the observations of patients who went through the sepsis screening, white blood cell count was the most prevalent SIRS criterion experienced in the ICU. In addition, 12.32% of the observations (when four measurements were available at the same time) showed a three-factored SIRS of tachypnea-high heart rate-high white blood cell count. It is critical to consider that there are 6 possible pairs of combinations, 4 possible trios of combinations, and 1 combination considering all the parameters as the possible sepsis scenario in the ICU. As mentioned above for qSOFA, no sepsis is another possible scenario besides these 11 possible SIRS scenarios in the ICU (which is also the case for our observations). Identifying the most prevalent criterion and sepsis scenario in the ICU for SIRS can help practitioners and researchers in the diagnosis, treatment, and design of further factorial experiments.

**Figure 6:**
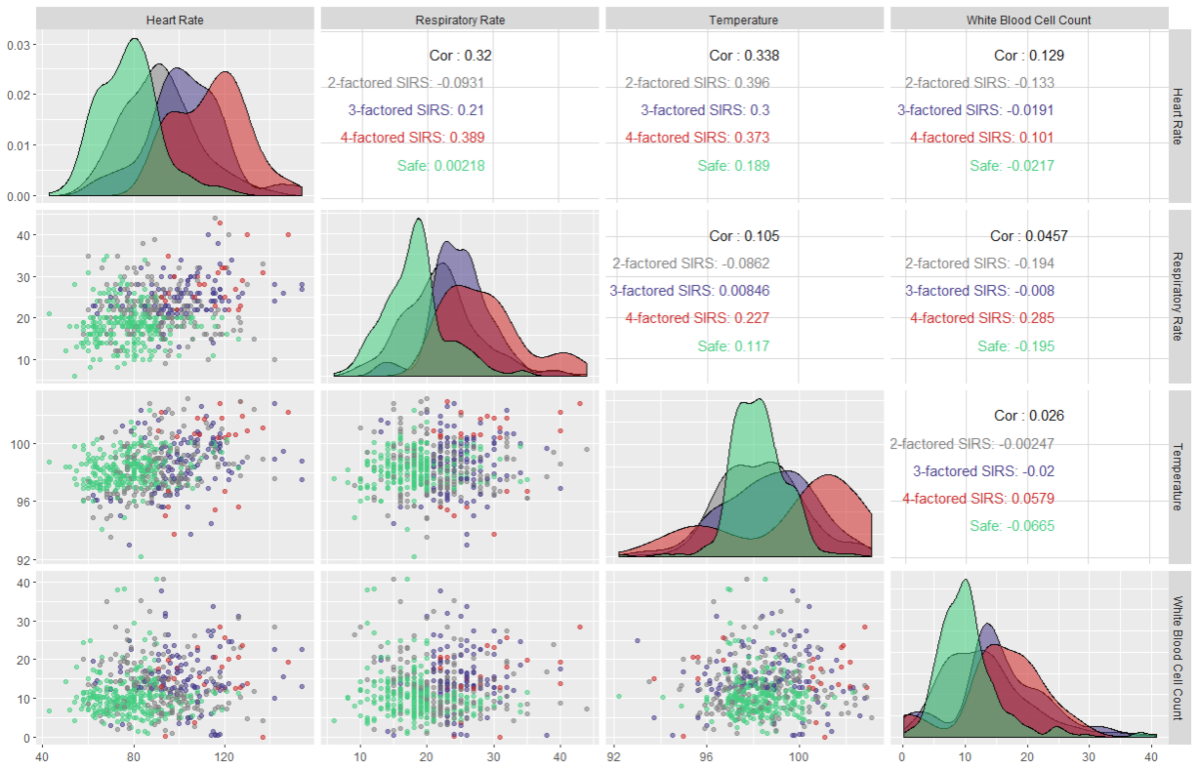
Facet grid illustration of sepsis-2 (SIRS) parameters to capture the underlying relationship between parameters and the most prevalent sepsis scenario in the intensive care unit. SIRS: systemic inflammatory response syndrome.

Figure 6 shows a facet grid plot of SIRS (sepsis-2) parameters to capture the most prevalent SIRS scenario and the underlying dichotomy among the parameters. The absolute Pearson correlation coefficients for heart rate-respiratory rate, heart rate-temperature, heart rate-white blood cell count, respiratory rate-temperature, respiratory rate-white blood cell count, and temperature-white blood cell count were 0.32, 0.34, 0.13, 0.11, 0.05, and 0.03, respectively. These insignificant absolute correlation coefficients invalidate the possibility of any correlation among the critical parameters, thereby ensuring that multicollinearity does not exist between the parameters and further advocates for the dichotomy among them. However, despite being not statistically significant, the absolute correlation coefficients were not negligible in the case of heart rate-respiratory rate and heart rate-temperature pairs. Understanding this relationship can help in developing predictive models as it implies that the overdetermined system involved in the modeling is a full-ranked matrix (ie, not rank-deficient). However, the lack of multicollinearity cannot guarantee that two random variables are statistically independent.

### Patients’ First Observation Only for qSOFA

**Figure 7:**
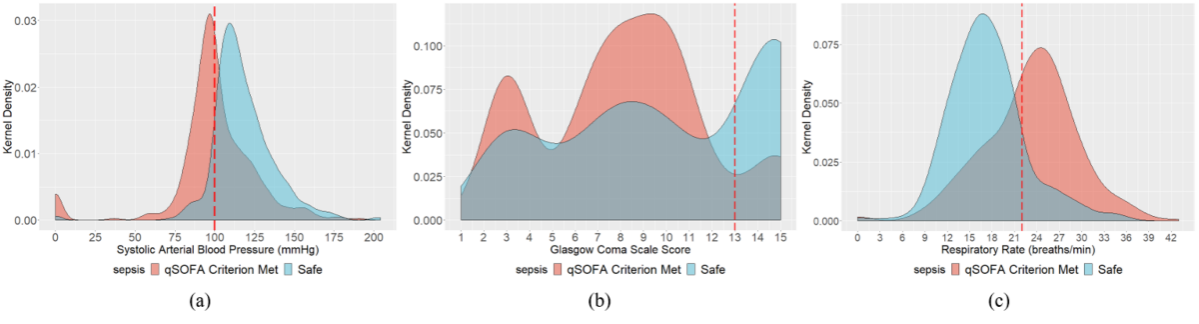
Kernel density estimation distribution of quick Sequential Organ Failure Assessment (qSOFA) parameters for both safe and qSOFA criterion–met patients at first observations to identify the prevalent qSOFA parameters.

In the second phase of this study, we dissected data for only the first observations of each hospital admission. This may address two possible selection biases, including the opportunity to test the intrageneralizability of the result of this observational study. First, it is intuitive that the longer the patient stays in the ICU, there will be more observations available for that particular patient. This may influence the results of our study to some extent if there is considerable disproportion between the length of stay among patients. Second, when evaluating the respiratory rate for ICU patients, there may be a possible blend in the data between patients under intubated breathing and those naturally breathing. The kernel density estimation distribution of qSOFA parameters for both safe and qSOFA criterion–met observations are presented in Figure 7 to understand the prevalent qSOFA parameters. We found that 32.58% of the systolic arterial blood pressure observations, 44.54% of the Glasgow Coma Scale measurements, and 40.53% of the respiratory rate observations met the respective qSOFA criterion. This observational study entirely relied on passive retrospective observation without assigning any further treatment. The results suggest that altered mental status is the most prevalent qSOFA criterion experienced in the ICU. In addition, 18.25% of the observations had a two-factored qSOFA of high respiratory rate and altered mental status (among 3C_3_+3C_2_=4 possibilities), resulting in this pair as the most prevalent qSOFA (sepsis-3) scenario in the ICU, although the no-sepsis scenario is also possible.

**Figure 8:**
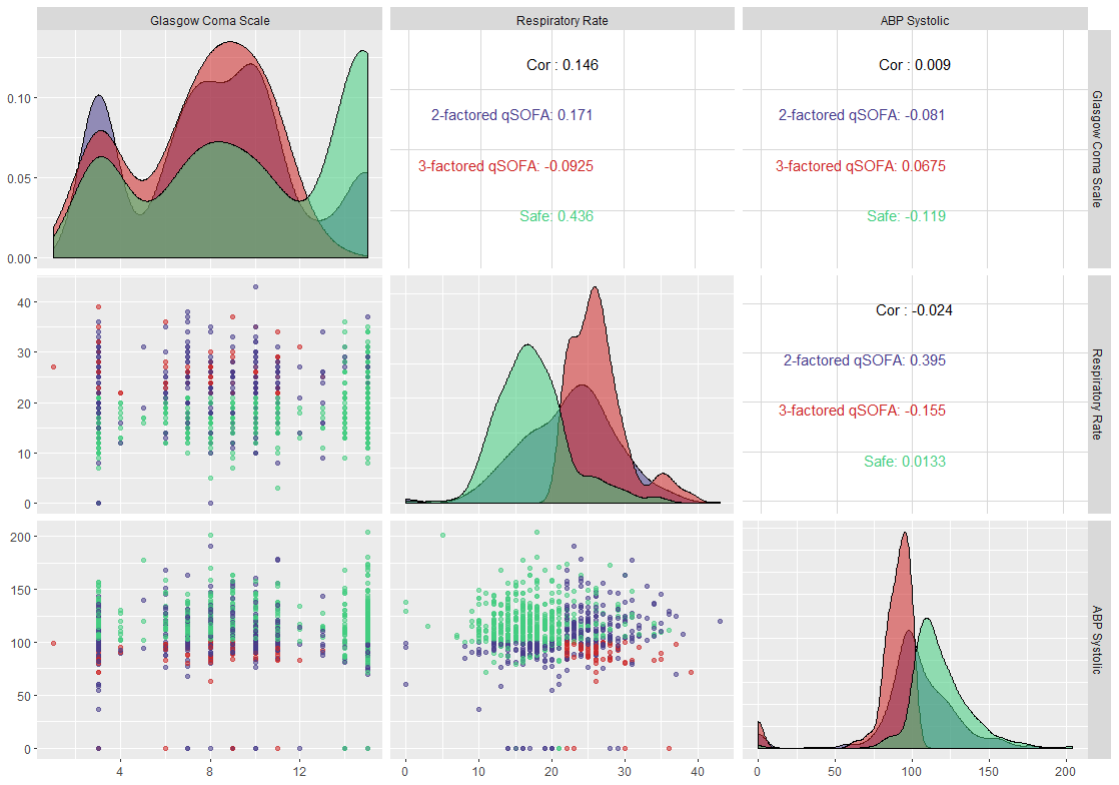
Facet grid illustration of sepsis-3 (qSOFA) parameters to capture the underlying relationship between parameters and the most prevalent sepsis scenario of patients at first observations in the intensive care unit. qSOFA: quick Sequential Organ Failure Assessment.

Figure 8 shows the facet grid on qSOFA parameters to understand the most prevalent qSOFA scenario and the underlying dichotomy among the parameters. The absolute Pearson correlation coefficients for respiratory rate-Glasgow Coma Scale measurement, Glasgow Coma Scale measurement-systolic arterial blood pressure, and respiratory rate-systolic arterial blood pressure pairs were 0.15, 0.01, and 0.02, respectively. These insignificant correlation coefficients invalidate the possibility of any correlation among the critical parameters, ensuring that multicollinearity does not exist between the parameters and further bolsters the dichotomy among them. However, the lack of multicollinearity cannot guarantee that two random variables are statistically independent.

### Patients’ First Observation Only for SIRS

**Figure 9:**
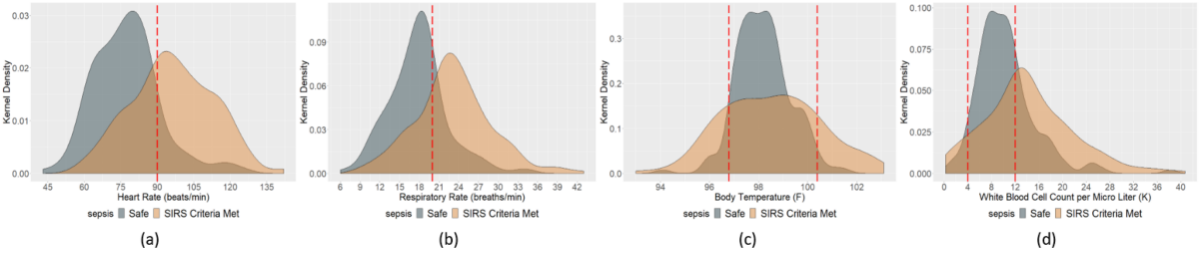
Kernel density estimation distribution of systemic inflammatory response syndrome (SIRS) parameters for both safe and sepsis criterion–met patients at first observations to identify the prevalent SIRS parameters.

Figure 9 shows the kernel density estimation distribution of SIRS parameters for both safe and SIRS criterion–met observations using only the first observations. We found that 57.03% of the heart rate observations, 45.89% of the respiratory rate observations, 33.93% of the body temperature observations, and 60.57% of the white blood cell count observations met the respective SIRS criterion. These results suggest that white blood cell count is the most prevalent criterion experienced in the ICU, albeit considering that both the white blood cell count and respiratory rate had significant prevalence. In addition, 11.38% of the SIRS criteria–met sepsis patients showed a three-factored SIRS of tachypnea-high heart rate-high white blood cell count (among 4C_4_+4C_3_+4C_2_=11 possibilities), resulting in this trio as the most prevalent sepsis (SIRS) scenario in the ICU. It is important to consider that there are 6 possible pairs of combinations, 4 possible trios of combinations, and 1 combination considering all of the parameters as the possible sepsis scenarios in the ICU, and that no sepsis is another possible scenario. Determining the most prevalent SIRS criterion and sepsis scenario at the first observation upon hospitalization can help practitioners and researchers in diagnosis, treatment, and further factorial experiments.

**Figure 10:**
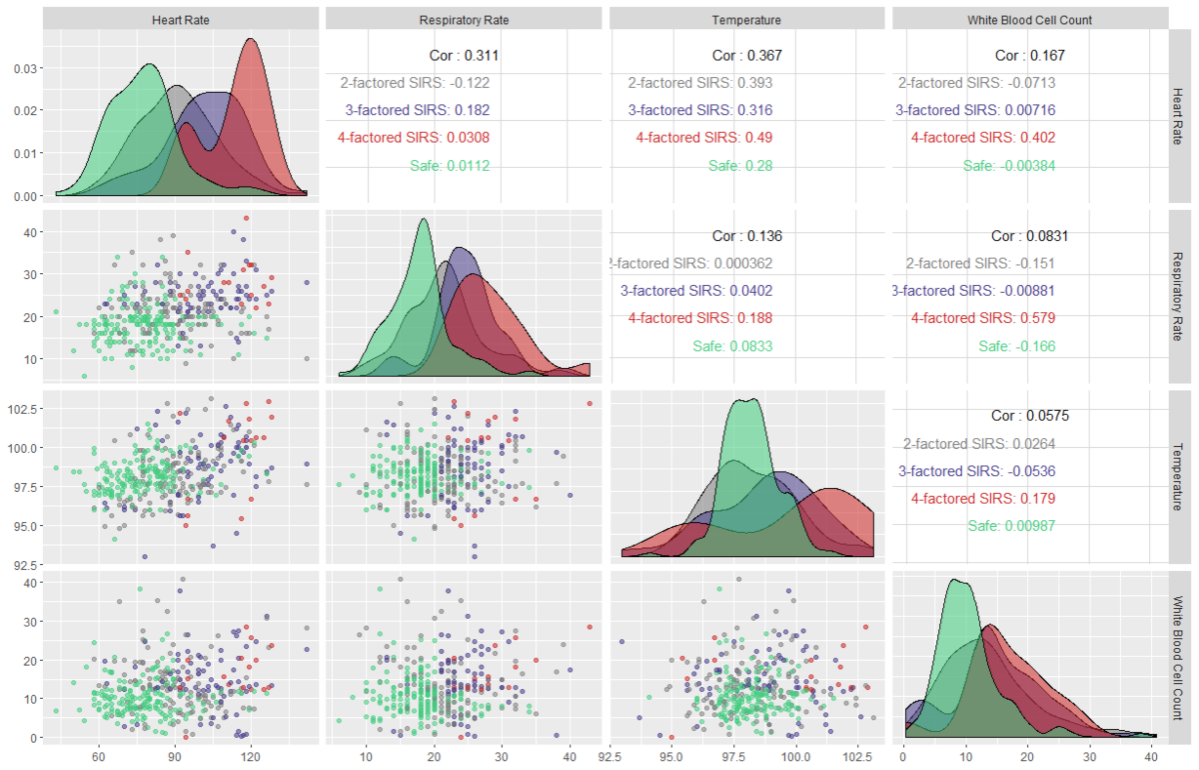
Facet grid illustration of sepsis-2 (SIRS) parameters to capture the underlying relationship between parameters and the most prevalent sepsis scenario of patients at first observations in the intensive care unit. SIRS: systemic inflammatory response syndrome.

Figure 10 shows the facet grid illustration for SIRS parameters at the first observation. The insignificant absolute Pearson correlation coefficients invalidate the possibility of any correlation among the critical parameters, thereby ensuring that multicollinearity does not exist between the parameters and further bolsters the dichotomy among them. However, similar to the case for all observations, the absolute correlation coefficients were not negligible in the case of heart rate-respiratory rate and heart rate-temperature pairs.

## Discussion

### Theoretical Reasoning

This study reveals that altered mental status and systolic arterial blood pressure are the most and least prevalent qSOFA criteria, respectively, observed in the ICU. Mathematically, blood pressure is the product of systemic vascular resistance and cardiac output. Hence, with the decrease in systemic vascular resistance due to vasodilation, blood pressure will drop down if the cardiac output remains the same. However, in practice, when the systemic vascular resistance drops down, the human body immediately tries to maintain the equilibrium for a few moments and compensates with the cardiac output. Cardiac output depends on the respiratory rate in a nonlinear and proportionate manner; hence, the increase in the respiratory rate increases the cardiac output and maintains the equilibrium of the blood pressure initially. However, over time, that equilibrium breaks down, although the cardiac output (and consequently respiratory rate) continually tries to reach a stable state. This fact advocates the possibility of respiratory rate to be a more prevalent criterion compared to systolic arterial blood pressure as a symptom. From the aspect of SIRS criteria, the reason for the white blood cell count to emerge as the most prevalent criterion is intuitive. When a microorganism invades, the body’s immune response is triggered and white blood cells appear immediately. Heart rate, respiratory rate, and temperature are consequential symptoms associated with an increase in white blood cells and the immune response. As sepsis is a spectrum disease, one predictor may influence another during disease development and progression to septic shock, although they are not linearly correlated. The findings of this observational study support the established pathophysiology of sepsis described in the literature.

### Research Opportunities

Although MIMIC-III is an extensive critical care database, it is a single-center database comprising critical care unit electronic health record data of Beth Israel Deaconess Medical Center in Boston. Regardless of the myriad amount of patient data, the findings that are valid for the Beth Israel Deaconess Medical Center in Boston may not be useful for other medical centers and critical care units. The epidemiology and treatment facilities vary among the hospitals, states, and infrastructures of countries. Epidemiology and treatment facilities have a significant impact on patient outcome, as well as on patients’ symptom distributives. On the flip side, this observational study entirely relied on passive retrospective observation, and the dynamics of the treatment and medicine advance with time and research. In addition, the prevalence of the physiological parameters, along with time and resource variability, may also affect the interrelation nature among parameters. The results may also vary if considering the analysis from an individual aspect. Although a collective analysis infers the dichotomy among parameters, there may be a possibility that data from even one patient show strong multicollinearity. Again, the parameters measured may vary according to the therapeutics undertaken in the ICU. For instance, the Glasgow Coma Scale score may become low due to sedation, catecholamines may be responsible for healthy blood pressure, or mechanical ventilation may affect the respiratory rate. Any predictive modeling and treatment plan should take this variability and uncertainty into account.

This uncertainty around generalizability opens up new research opportunities in the health informatics domain in three possible directions: (1) Does this finding hold its generalizability while integrating data from multiple electronic health records? (2) How can we study confounding variables induced by numerous groups of people with different characteristics? (3) How can these findings address the confounding medical interventions in sepsis treatment?

Moreover, the comparison between qSOFA and SIRS can be extended to comparing SOFA and qSOFA, SIRS and SOFA, or all the three criteria available to better understand the underlying interrelations between the parameters.

### Conclusion

This study indicates that altered mental status (as assessed with the Glasgow Coma Scale) is the most prevalent qSOFA criterion and white blood cell count is the most prevalent SIRS criterion for patients in the ICU. Besides, two-factored sepsis comprising altered mental status and high respiratory rate (≥22 breaths/minute) is the most prevalent sepsis-3 (qSOFA) scenario, and two-factored sepsis of white blood cells and tachypnea is the most prevalent sepsis-2 (SIRS) scenario confronted in the ICU among patients screened for sepsis. In addition, the Pearson correlation coefficients advocate for the dichotomy among the sepsis parameters (for both qSOFA and SIRS). This study implies that sepsis diagnosis and treatment should be pertinent to its type, and in this regard, these multifactored attributes should be taken into account. Machine-learning predictive models should consider the most prevalent criterion pair, which would allow for a faster diagnosis. Moreover, the reasoning backed by the sepsis pathophysiology assures the interpretability that these results require. These findings can help obtain a better understanding of the algorithmic, as well as contextual challenges that influence predictive decisions in the ICU.

## Abbreviations

ICU: intensive care unit
MIMIC-III: Medical Information Mart for Intensive Care
qSOFA: quick Sequential Organ Failure Assessment
SIRS: systematic inflammatory response syndrome
SOFA: Sepsis-related Organ Failure Assessment
